# MicroRNA-377-3p exacerbates chronic obstructive pulmonary disease through suppressing ZFP36L1 expression and inducing lung fibroblast senescence

**DOI:** 10.1186/s12931-024-02696-3

**Published:** 2024-02-05

**Authors:** Fang Lu, Li-peng Yao, Dan-dan Gao, Tahereh Alinejad, Xin-qing Jiang, Qi Wu, Qiao-cheng Zhai, Ming Liu, Sheng-mei Zhu, Mao-xiang Qian, Li-feng Xu, Cheng-shui Chen, Feng Zhang

**Affiliations:** 1 Department of Respiratory and Critical Care, Quzhou Affiliated Hospital of Wenzhou Medical University, Quzhou, 324000 China; 2 Wenzhou Medical University, Wenzhou, 325035 China; 3 Joint Innovation Center for Engineering in Medicine, Quzhou Affiliated Hospital of Wenzhou Medical University, Quzhou, 324000 China; 4 The Key Laboratory of Interventional Pulmonology of Zhejiang Province, Department of Pulmonary and Critical Care Medicine, Centre of Precision Medicine, The First Affiliated Hospital of Wenzhou Medical University, Wenzhou, 325015 China; 5 Zhejiang Chinese Medical University the 2 nd Clinical Medical College, Hangzhou, 310053 China; 6 Institute of Pediatrics, Department of Hematology and Oncology, Institutes of Biomedical Sciences, National Children’s Medical Center, Children’s Hospital of Fudan University, Fudan University, Shanghai, 200032 China; 7 Ningbo College of Health Sciences, Ningbo, 315000 China; 8 Oujiang Laboratory (Zhejiang Lab for Regenerative Medicine, Vision, and Brain Health), School of Pharmaceutical Sciences, Wenzhou Medical University, Wenzhou, 325015 China

**Keywords:** COPD, miR-377-3p, Senescence, ZFP36L1, Lung fibroblast

## Abstract

Chronic obstructive pulmonary disease (COPD) is a leading aging related cause of global mortality. Small airway narrowing is recognized as an early and significant factor for COPD development. Senescent fibroblasts were observed to accumulate in lung of COPD patients and promote COPD progression through aberrant extracellular matrix (ECM) deposition and senescence-associated secretory phenotype (SASP). On the basis of our previous study, we further investigated the the causes for the increased levels of miR-377-3p in the blood of COPD patients, as well as its regulatory function in the pathological progression of COPD. We found that the majority of up-regulated miR-377-3p was localized in lung fibroblasts. Inhibition of miR-377-3p improved chronic smoking-induced COPD in mice. Mechanistically, miR-377-3p promoted senescence of lung fibroblasts, while knockdown of miR-377-3p attenuated bleomycin-induced senescence in lung fibroblasts. We also identified ZFP36L1 as a direct target for miR-377-3p that likely mediated its pro senescence activity in lung fibroblasts. Our data reveal that miR-377-3p is crucial for COPD pathogenesis, and may serve as a potential target for COPD therapy.

**Supplementary Information:**

The online version contains supplementary material available at 10.1186/s12931-024-02696-3.

## Introduction

Chronic obstructive pulmonary disease (COPD) is characterized by a variable extent and combination of small airway disease and emphysema. It is a major and still growing cause of death worldwide [1, 2]. Narrowing of the small airways is recognized as an early and important mechanism for COPD progression [3]. Although the underlying mechanisms and cells driving the development of this pathology are not fully understood, fibroblasts are considered as an crucial cell type in repair and produce growth factors, cytokines, extracellular matrix (ECM) and metalloproteinases to remodel the airway.

Accelerated lung ageing has been proposed to have a role in the pathophysiology of COPD [4, 5]. Several mechanisms of accelerated ageing have been found in COPD with cellular senescence being the most thoroughly characterized to be enhanced in lung tissues from COPD patients [6]. Cellular senescence is a state of irreversible cell cycle arrest, but with a metabolic and secretory program that is referred to as the senescence-associated secretory phenotype (SASP) [7]. The key features of senescence encompass the initiation of cell-cycle regulatory pathways, namely p16, p53 and p21, as well as the manifestation of senescence-associated β-galactosidase (SA-β-gal) activity [8]. COPD-derived fibroblasts have increased cellular senescence. The accumulation of senescent fibroblasts promote COPD progression through aberrant ECM deposition and SASP [6].

MicroRNAs (miRNAs) are small noncoding RNAs with 20–25 nucleotides in length. MiRNAs regulate gene expression in multicellular organisms by affecting both the stability and translation of mRNAs. They target the 3’-UTR of mRNA transcripts via complementary sequences and repress the gene expression at post-transcriptional level [9, 10]. The deregulation of miRNAs has been closely related to a variety of pathogenesis including COPD. miR-24-3p is decreased in COPD. Inhibition of miR-24-3p increases alveolar type II epithelial cell apoptosis, and emphysema severity. As a regulator of the cellular response to DNA damage, miR-24-3p suppresses apoptosis through BIM and suppressed homology-directed DNA repair via BRCA1 [11]. The expression of miR-21 is up-regulated in human lung tissue and is correlated with reduced lung function in COPD. Inhibition of miR-21 reduces airway macrophages, neutrophils, and lymphocytes, and improves lung function through SATB1/S100A9/NF-κB axis in mouse models of COPD [12]. Although these studies have led to an understanding of the role of miRNAs in COPD progression, our understanding of their role in COPD is still quite limited compared to miRNAs in tumors.

MiR-377-3p is a novel tumor regulatory miRNA whose biological functions are wildly unclear. MiR-377-3p has been shown to possess tumor-inhibiting effects in clear cell renal cell carcinoma and hepatocellular carcinoma [13, 14]. In addition, it was reported to inhibit cell metastasis and epithelial-mesenchymal transition in cervical carcinoma through targeting SGK3 [15]. Recent study showed that miR-377-3p downregulated EGR1 and promoted benzo[a]pyrene-induced lung tumorigenesis by Wnt/β-Catenin transduction [16]. Based on a COPD population cohort study, we found that miR-377-3p is increased in blood of COPD patients compared to normal counterparts. However, the contribution and the pathogenic mechanism of miR-377-3p in COPD is largely unknown.

In this study, we found that miR-377-3p was up-regulated in COPD patients, with a majority of its localization in lung fibroblasts. Inhibition of miR-377-3p improved chronic smoking-induced COPD in mice. Mechanistically, miR-377-3p promoted senescence of lung fibroblasts, while knockdown of miR-377-3p attenuated bleomycin-induced senescence in lung fibroblasts. We also identified ZFP36L1 as a direct target for miR-377-3p that likely mediated its pro senescence activity in lung fibroblasts. Our data reveal that miR-377-3p is crucial for COPD pathegenesis, and may serve as a potential target for COPD therapy.

## Materials and methods

### Induction of experimental COPD

Male WT C57BL/6 mice (8–10 week old) were purchased from Vital River Laboratory Animal Technology (Beijing, China) and kept under sterile conditions (following a 12 h light/dark cycle), with 1-week acclimatization period. Mice were chronically exposed to cigarette smoking from the cigarettes (Chinese Lion), or normal air, via the nose only for 75 min at a time, twice per day, 5 days per week 24 weeks to induce experimental COPD. The body weights were measured weekly. For miRNA antagomir intervention, mice were administered with miR-377-3p specific or scrambled antagomir intranasally once a week (2.5 mg/kg) under isoflurane anesthesia. The protocols for animal experiments have been reviewed and approved by Laboratory Animal Management and Ethics Committee of Hangzhou Medical College in Zhejiang Province (license number of animal use permit: SYXK 2023-0011, license number of approval of animal ethical and welfare: ZJCLA-IACUC-202,308).

### Cell culture

Human lung fibroblast MRC-5 and 293T cells was purchased from Cell Bank of the Chinese Academy of Sciences (Shanghai, China) and was cultured in DMEM containing 10% FBS and antibiotics in 5% CO_2_ at 37℃ in a humidified atmosphere. For induction of cellular senescence, MRC-5 cells were treated with bleomycin (50 µg/mL) for 12 h and cultured in fresh medium for 2 days.

### Isolation of primary lung fibroblasts and epithelial cells

Primary human or mouse lung fibroblasts and lung epithelial cells were isolated as described previously [17]. Lung tissues were cut into small pieces and incubated with digestion buffer (0.1% collagenase, 0.05% trypsin, and 100 mg/mL DNase in Hanks’ balanced salt solution) for 1 h at 37℃. The digested tissue suspensions were filtered through a 40 μm cell strainer, centrifuged at 500 × g for 5 min and pellets were collected. After the lysis of red blood cells, the cells were resuspended and incubated for 1 h with biotin-conjugated anti-CD16/32, anti-CD45, and anti-CD31 antibodies. The cells were then rinsed, resuspended, and incubated for 30 min with streptavidin magnetic beads. Tubes containing the incubated cells were then applied to a magnet to deplete endothelial cells, lymphocytes, monocytes/macrophages, natural killer (NK) cells, neutrophils, and other haematopoietic cells. Supernatants were collected and plated into tissue culture plates. After 1 h incubation at 37℃, the suspended lung epithelial cells were harvested for experiments. The adherent lung fibroblasts were grown in MEM media supplemented with 10% FBS. Fibroblasts at passage 3–5 were used for experiments.

### Human subjects

Normal and COPD lung tissue specimens were obtained from patients who underwent lobectomy or pneumonectomy for lung cancer in the Quzhou Affiliated Hospital of Wenzhou Medical University. COPD was diagnosed based on a combination of medical history and physical examination including pulmonary function test using spirometry. Informed written consent was obtained from all participants. All studies have been approved by the Institutional Review Board at the Quzhou Affiliated Hospital of Wenzhou Medical University.

### Plasmids, transfection and lentiviral infection

MiR-377-3p agomir (#HY-R00842A) and antagomir (#HY-RI00842A) were purchased from MedChemExpress LLC (Shanghai, China). Compared with common mimics/inhibitors, miRNA agomir/antagomir has higher stability and inhibitory effect in animal experiments, and is more likely to pass through cell membranes and tissue interstitial space and be enriched in target cells. Cells were transfected in 6-well plates using RNAiMAX transfection reagent (Life Technologies, Thermo Fisher Scientific) and OptiMEM media according to manufacturer’s instruction.

For ZFP36L1 expression in MRC-5 cells, Flag-tagged ZFP36L1 vector was purchased from Sino Biological (#HG19776-CF). The ZFP36L1 cDNA ORF was then subcloned into pCDH-CMV-puro lentiviral expression vector. The method for lentivirus production and transduction was introduced previously [18].

ZFP36L1 miRNA 3’-UTR clone (#MiUTR3H-03855) in pMirTarget 3’-UTR Assay Vector was ordered from Creative Biogene (NY, USA). Mutated 3’ UTR of ZFP36L1 was generated using the Hieff Mut™ Site-Directed Mutagenesis Kit (YEASEN, #11003ES10). Lipofectamine 3000 reagents were performed for transfection according to the manufacturer’s instruction (Invitrogen).

### Real-time PCR

The extraction of total RNA was performed utilizing Trizol reagent in conjunction with the conventional liquid phase method incorporating chloroform. The RNA sample underwent reverse transcription to generate complementary DNA (cDNA) utilizing the RT-Mix kit (Takara, #RR036A) with either random primer or gene specific RT primer. The real time PCR experiment was conducted using the SGExcel FastSYBR Master premix (Sangon Biotech, #B532955-0005) following standard reaction conditions. The primers employed in this experiment are documented in Table 1. The relative expression level of the target gene or miRNA was determined utilising the 2^−ΔΔCt^ methodology, with β-actin or U6 serving as the internal reference gene.

**Table 1:** Sequence of primers used for miRNA agomir, antagomir and qRT-PCR

Primer name	Sequences (5’→3’)	Species	Application
miR-377-3p agomir	AUCACACAAAGGCAACUUUUGU	human	overexpression
miR-377-3p antagomir	ACAAAAGUUGCCUUUGUGUG	human	inhibition
miR-377-3p RT	GTCGTATCCAGTGCAGGGTCCGAGGTATTCGCACTGGATACGACacaaaag	human/mouse	reverse-transcription
U6 RT	AACGCTTCACGAATTTGCGT	human/mouse	reverse-transcription
miR-377-3p F	ATCACACAAAGGCAACTTTTGT	human/mouse	real-time PCR
miR-377-3p R	CCAGTGCAGGGTCCGAGGTAT	human/mouse	real-time PCR
U6 F	CTCGCTTCGGCAGCACA	human/mouse	real-time PCR
U6 R	AACGCTTCACGAATTTGCGT	human/mouse	real-time PCR
ZFP36L1 F	AGCGAAGTTTTATGCAAGGGTAAC	human	real-time PCR
ZFP36L1 R	CTTTCTGTCCAGCAGGCAAC	human	real-time PCR
CDKN1A (p21) F	CGTGTCACTGTCTTGTACCCT	human	real-time PCR
CDKN1A (p21) R	GCGTTTGGAGTGGTAGAAATCT	human	real-time PCR
p53 F	CAGCACATGACGGAGGTTGT	human	real-time PCR
p53 R	TCATCCAAATACTCCACACGC	human	real-time PCR
IL-1β F	TGCACGCTCCGGGACTCACA	human	real-time PCR
IL-1β R	CATGGAGAACACCACTTGTTGCTCC	human	real-time PCR
IL-6 F	CCAGGAGCCCAGCTATGAAC	human	real-time PCR
IL-6 R	CCCAGGGAGAAGGCAACTG	human	real-time PCR
IL-8 F	GAGTGGACCACACTGCGCCA	human	real-time PCR
IL-8 R	TCCACAACCCTCTGCACCCAGT	human	real-time PCR
MCP-1 F	AGCTCGCACTCTCGCCTCCAG	human	real-time PCR
MCP-1 R	GGCATTGATTGCATCTGGCTGAGC	human	real-time PCR
MMP9 F	TGTACCGCTATGGTTACACTCG	human	real-time PCR
MMP9 R	GGCAGGGACAGTTGCTTCT	human	real-time PCR
PAI-1 F	CCACTGGAAAGGCAACATGACCAGG	human	real-time PCR
PAI-1 R	GCCATGCGGGCTGAGACTATGACAG	human	real-time PCR
CCL20 F	GGCGAATCAGAAGCAGCAAGCAAC	human	real-time PCR
CCL20 R	ATTGGCCAGCTGCCGTGTGAA	human	real-time PCR
β-actin F	AGAGCTACGAGCTGCCTGAC	human	real-time PCR
β-actin R	AGCACTGTGTTGGCGTACAG	human	real-time PCR

### ELISA

The levels of TNFα and CXCL1 proteins in mouse lung tissue homogenates were quantified using anti-mouse ELISA kits ordered from R&D Systems. The measurements were conducted according to the manufacturer’s instructions. To ensure consistency, the obtained values were normalized to the total protein content as previously described [12]. To prepare human lung tissue homogenates, the lung tissues were rapidly frozen and homogenized on ice in RIPA buffer (Beyotime, #R0010) with phenylmethylsulfonyl fuoride (PMSF). Homogenates were sonicated (3 times in 5 s), centrifuged (500 g, 10 min at 4℃), and the central layer was collected. Total protein concentrations were determined using a BCA Protein Assay Kit (Thermo Fisher Scientific Pierce). RIPA buffer was used to dilute lung tissue homogenates to a final protein concentration of 500 mg/mL.

### Pulmonary function assessment

Pulmonary function tests were performed in a forced pulmonary maneuver system (Shanghai Yuyan Instrument Co., Ltd.) according to manufacturer’s instruction and previous protocols [19]. Mice were anesthetized with ketamine (100 mg/kg) and xylazine (10 mg/kg), and their tracheas were cannulated. All maneuvers were performed at least three times, and the average was calculated.

### Western blotting

Cells were washed with 1× PBS and lysed in 1× SDS buffer containing protease inhibitors. The lysate was collected, boiled at 100 °C for 10 min, and centrifuged to remove cell debris. Equal amounts of protein were separated by SDS-PAGE and transferred onto a PVDF membrane. The membrane was blocked with 0.1% casein at room temperature for 60 min and then incubated overnight at 4 °C with the corresponding primary antibodies, including p53 (Abcam, #ab26), p21 (Abcam, #ab109199), p16 (Abcam, #ab109349), β-actin (Cell Signaling Technology, #3700). The membrane was then incubated with an HRP-conjugated secondary antibody at room temperature for approximately 60 min, followed by detection of the protein bands using an ECL protein blotting substrate.

### SA-β-gal staining

The SA-β-gal staining kit (Beyotime, #C0602) was used according to the standard instructions to detect SA-β-gal activity and determine senescent cells. Tissue sections were counterstained with eosin to facilitate observation. The SA-β-gal-positive cells was calculated using ImageJ after imaging with a Nikon inverted microscope.

### EdU immunofluorescence

The EdU-488 cell proliferation detection kit (Beyotime, #C0071) was used according to the standard instructions. Briefly, the prepared EdU working solution (20 µM) was added to a 6-well plate in an equal volume, resulting in a final concentration of 10 µM. The cells were then incubated for an additional 2 h. The cells were fixed, washed, permeabilized, and stained sequentially, and the percentage of EdU-positive cells was calculated using ImageJ after imaging with a Nikon inverted fluorescence microscope.

### Luciferase reporter assay

The recombinant vector containing either wild-type (WT) or mutated (MT) 3’-UTR of ZFP36L1 was transfected into HEK 293T cells or primary fibroblasts using Lipofectamine 3000. miRNA agomirs were subsequently transfected into cells depends upon the performance of experiments. The luciferase activity was measured 24 h after transfection.

### Assessment of alveolar enlargement

The lung tissue samples were fixed with 4% paraformaldehyde, embedded in paraffin and sectioned (3-µm thick). Longitudinal sections of the left single-lobe lung were stained with hematoxylin and eosin. Alveolar enlargement was assessed by measuring the average alveolar diameter in 10 viable images (magnification of ×40) using the mean linear intercept method as described previously [12].

### Bronchoalveolar lavage

Airway inflammation was assessed by differential enumeration of inflammatory cells in bronchoalveolar lavage fluid (BALF) as described previously [20]. Briefly, bronchoalveolar lavage fluid was obtained using a 20 G intravenous catheter inserted into the trachea with 1 mL PBS. After centrifugation at 1,000 g for 3 min, the pellets were resuspended in 1 mL PBS. A hemocytometer and a Diff-Quick stain kit (Sysmex, Kobe, Japan) were used to assess total and differential cell counts.

### Statistical analysis

Data is presented as mean ± SD. Statistical analyses were performed using GraphPad Prism 7.0 (GraphPad Software, La Jolla California USA). Differences between the groups were analyzed using Student’s t-test or one-way ANOVA followed by Dunnett’s multiple comparisons test. *p* < 0.05 was considered as significance.

## Results

### Mir-377-3p is up-regulated in COPD patients and chronic smoking induced mice

**Fig. 1:**
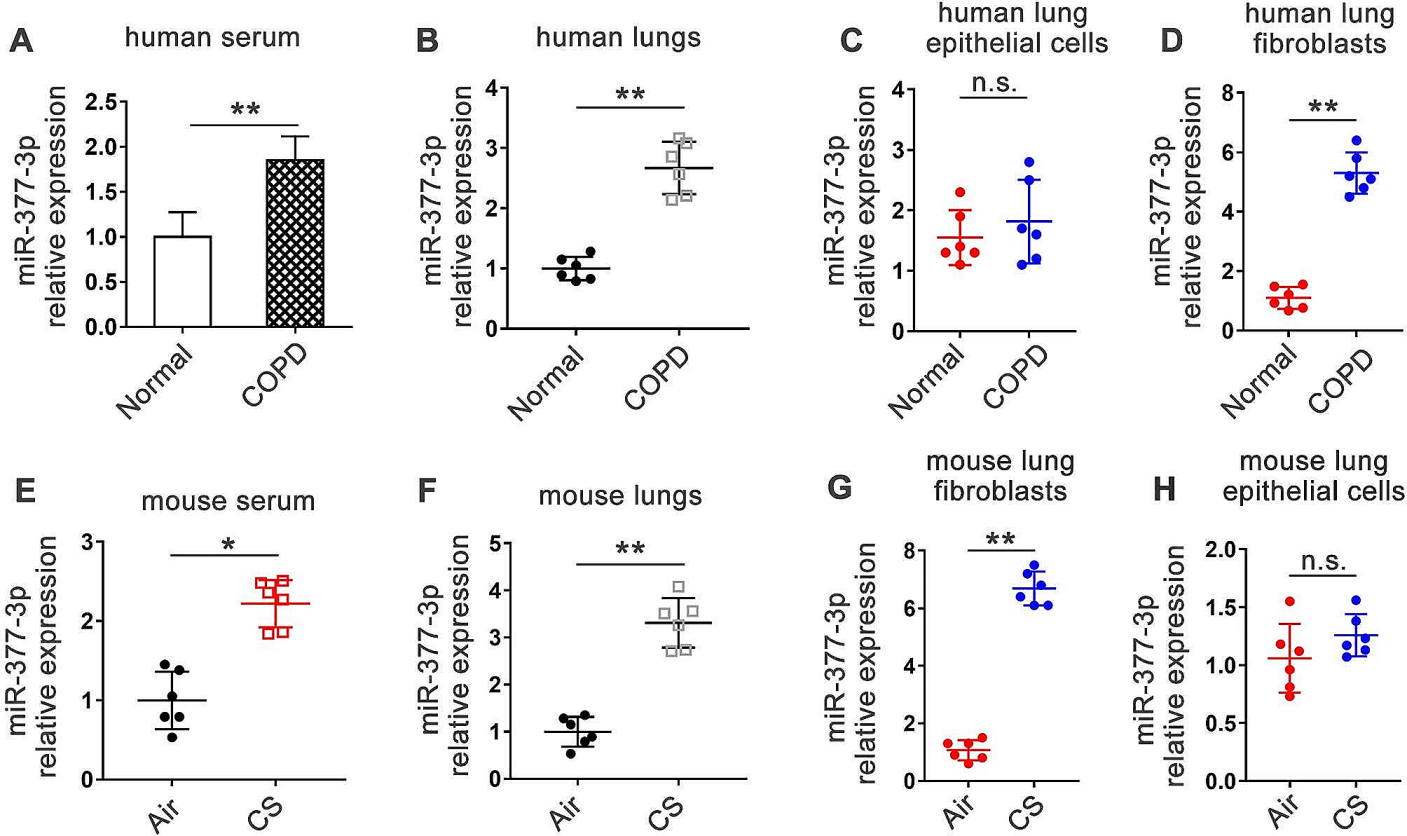
MiR-377-3p is increased in COPD. **(A)** The abundance of miR-377-3p in blood samples from normal people and COPD patients (*n* = 6 per group). **(B)** The expression of miR-377-3p in human lung samples (*n* = 6 per group). **(C-D)** The expression of miR-377-3p in isolated primary human lung epithelial cells and lung fibroblasts. **(E)** The abundance of miR-377-3p in blood samples from mice of control (Air) and experimental COPD model (CS). **(F)** MiR-377-3p expression in mouse lung samples. **(G-H)** The expression of miR-377-3p in isolated primary mouse lung fibroblasts and epithelial cells. Data are presented as means ± SD. n.s., not significant. *p < 0.05; **p < 0.01

Firstly, we performed real-time PCR assay to confirm the upregulation of miR-377-3p in the blood and lung samples from COPD patients (Fig. 1A and B). Furthermore, the expression of miR-377-3p was significantly higher in lung fibroblasts of patients with COPD than in normal subjects, but in lung epithelial cells, the expression of miR-377-3p did not differ significantly between patients with COPD and normal subjects (Fig. 1C and D). In order to determine whether the alteration is conserved across species, we examined the abundance of miR-377-3p in serum and lungs of mice. We found that chronic smoking (CS) induced COPD mice had significantly higher levels of miR-377-3p in serum and lung than normally ventilated control mice, which was similar to our observation in the human population (Fig. 1E and F). Notably, in isolated primary mouse lung fibroblasts, miR-377-3p expression in CS-treated mice was 6.3-fold higher than that in the normally ventilated group (Fig. 1G), whereas the ratio was 3.3 in the whole lung tissue of mice (Fig. 1F), and the difference between control and CS model mice in isolated mouse lung epithelial cells was not significant (Fig. 1H), suggesting that the up-regulation of miR-377-3p expression in the lungs of mice with COPD may be mainly derived from lung fibroblasts.

### Inhibition of mir-377-3p protects mice from experimental COPD

**Fig. 2:**
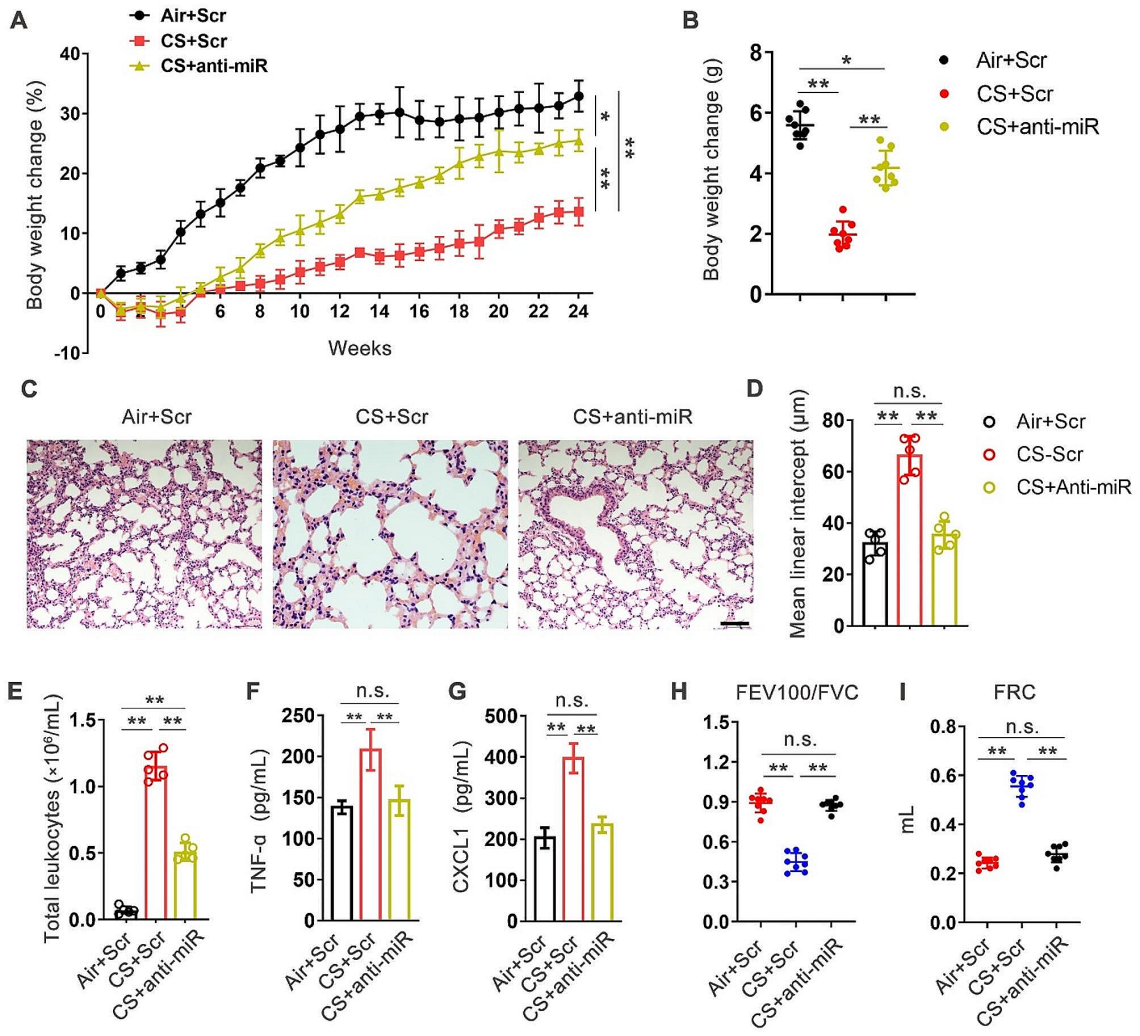
Down-regulation of miR-377-3p protects mice from chronic smoking induced experimental COPD. C57BL/6 mice (8–10 wk old) were normally ventilated (Air) or exposed to chronic smoking (CS) for 24 weeks to induce experimental COPD. miR-377-3p specific antagomir (anti-miR) or scrambled (Scr) antagomir were treated intranasally weekly. **(A)** Percentage of body weight change was measured throughout the 24-week period. **(B)** Body weight change. **(C)** Histological staining of lung. Scale bar, 80 μm. **(D)** The mean linear intercept of lung tissues calculated based on histopathology of (C). **(E)** The change of total leukocytes in BALF. **(F, G)** TNF-ɑ and CXCL1 proteins in whole-lung homogenates. Pulmonary function, including FEV100/FVC **(H)** and forced residual capacity (FRC) **(I)** were measured (*n* = 8 mice per group). Data are presented as means ± SD. n.s., not significant. *p < 0.05; **p < 0.01

To further characterize the role of miR-377-3p in COPD pathogenesis, we constructed experimental COPD mice by chronic smoking exposure. As shown in Fig. 2A and B, CS exposure limited body weight gain, while inhibition of miR-377-3p using antagomir alleviated the limitation. Histopathologically, inhibition of miR-377-3p significantly ameliorated the destruction of lung parenchymal tissues induced by cigarette smoking (Fig. 2C and D). Regarding lung inflammation, suppression of miR-377-3p reduced the number of inflammatory cells, including total leukocytes, macrophage, neutrophils and lymphocytes, in alveolar lavage fluid (Fig. 2E and S1). CS increased the expression of inflammatory cytokines and chemokines in lung tissues, which showed a significant regression after miR-377-3p inhibition (Fig. 2F and G). In terms of lung function, miR-377-3p inhibition considerably reduced pulmonary dysfunction caused by cigarette smoking (Fig. 2H, I and S2). These results suggest that miR-377-3p is not only a biomarker in the blood of COPD patients, but that it also play an important role in COPD pathology.

### Ectopic expression of mir-377-3p induces cellular senescence in lung fibroblasts

**Fig. 3:**
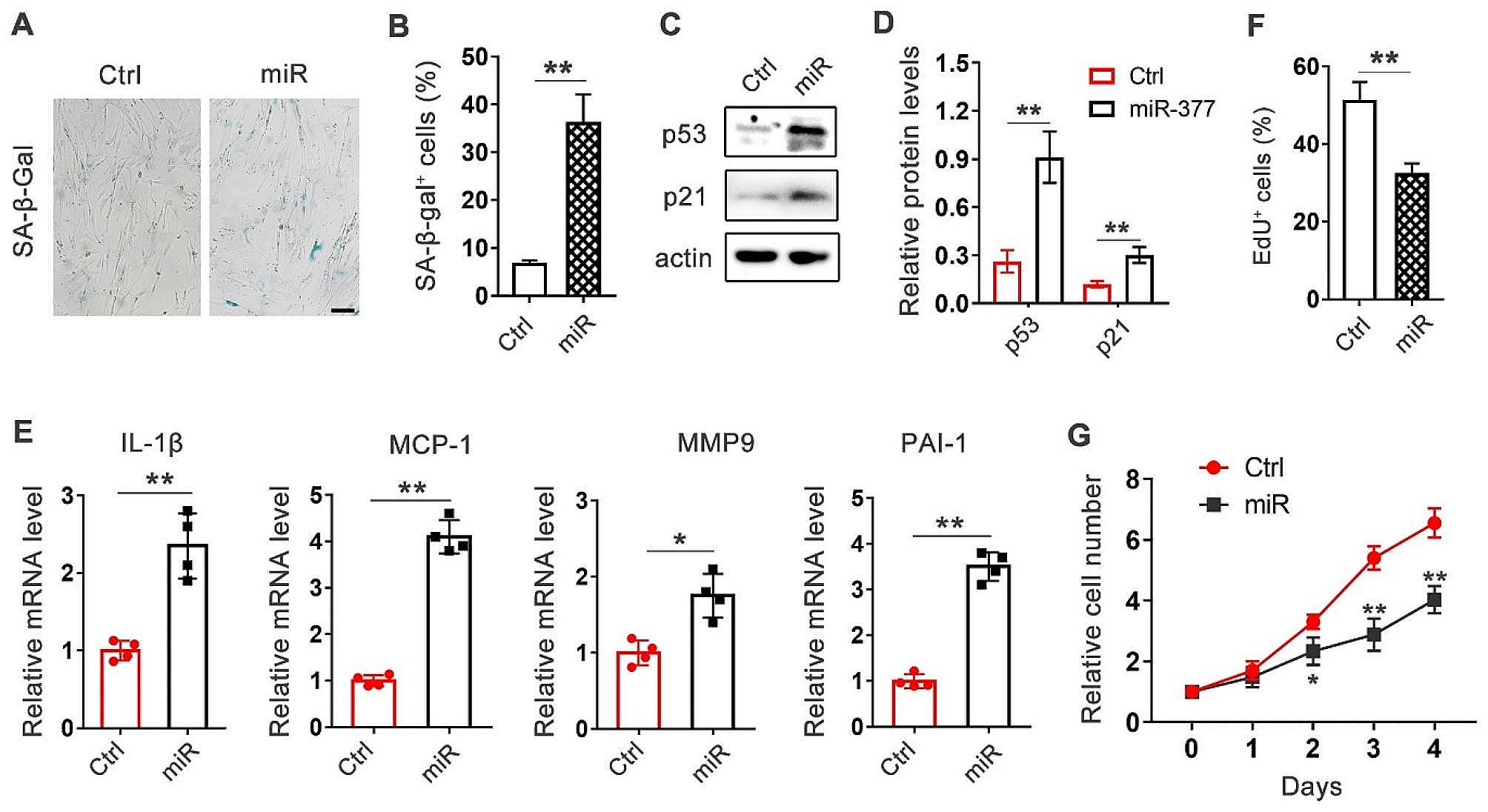
MiR-377-3p promotes cellular senescence in lung fibroblasts. Human lung fibroblasts MRC-5 were transfected with 50 nM control oligos or miR-377-3p agomir. Three days after transfection, cellular senescence was measured. **(A, B)** The senescence-associated β-galactosidase (SA-β-gal) activity in the cells were determined by staining. Scale bar, 100 µm. **(C, D)** The protein levels of p53 and p21 were determined by western blotting (n = 3). The intensity of bands was quantified by ImageJ software. **(E)** Levels of IL-1β, MCP-1, MMP9, PAI-1 were determined by real-time PCR (n = 4). **(F)** The cells were incubated with medium containing 5-ethynyl-2’-deoxyuridine (EdU) for 6 h. The quantification of EdU incorporation was assessed using a microscope at a magnification of 200×, with a minimum observation of 10 fields. **(G)** Cells in 24-well plates were transfected with control or miR-377-3p agomir. Cells were collected and counted at the indicated time points (*n* = 3). Data are presented as means ± SD. *p < 0.05; **p < 0.01

COPD is associated with cellular senescence [21]. To explore the mechanisms by which miR-377-3p influences COPD progression, we ectopicly expressed miR-377-3p in human lung fibroblasts MRC-5. We found miR-377-3p overexpression significantly induced cellular senescence indicated by the senescence-associated β-galactosidase (SA-β-gal) activity (Fig. 3A and B). p53/p21 is activated in response to DNA damage, leading to cellular senescence [22]. The protein levels of p53 and p21 were determined by western blotting. The result showed that the abundance of both p53 and p21 was increased after miR-377-3p overexpression, implying miR-377-3p activated DNA damage response signaling (Fig. 3C and D). Senescent lung fibroblasts exhibit a pro-fibrotic phenotype, secreting higher levels of senescence-associated secretory phenotype (SASP) proteins and promotes airway remodeling in COPD [6]. We found the mRNA expression of several SASP molecules, such as IL-1β, MCP-1, MMP9 and PAI-1, were prominently elevated upon miR-377-3p expression (Fig. 3E). Moreover, miR-377-3p also reduced fibroblast proliferation revealing by evaluating EdU incorporation and cell growth curve (Fig. 3F and G). Collectively, these results supported that up-regulation of miR-377-3p induced cellular senescence in lung fibroblasts.

### Suppression of mir-377-3p alleviates cellular senescence in lung fibroblasts

**Fig. 4:**
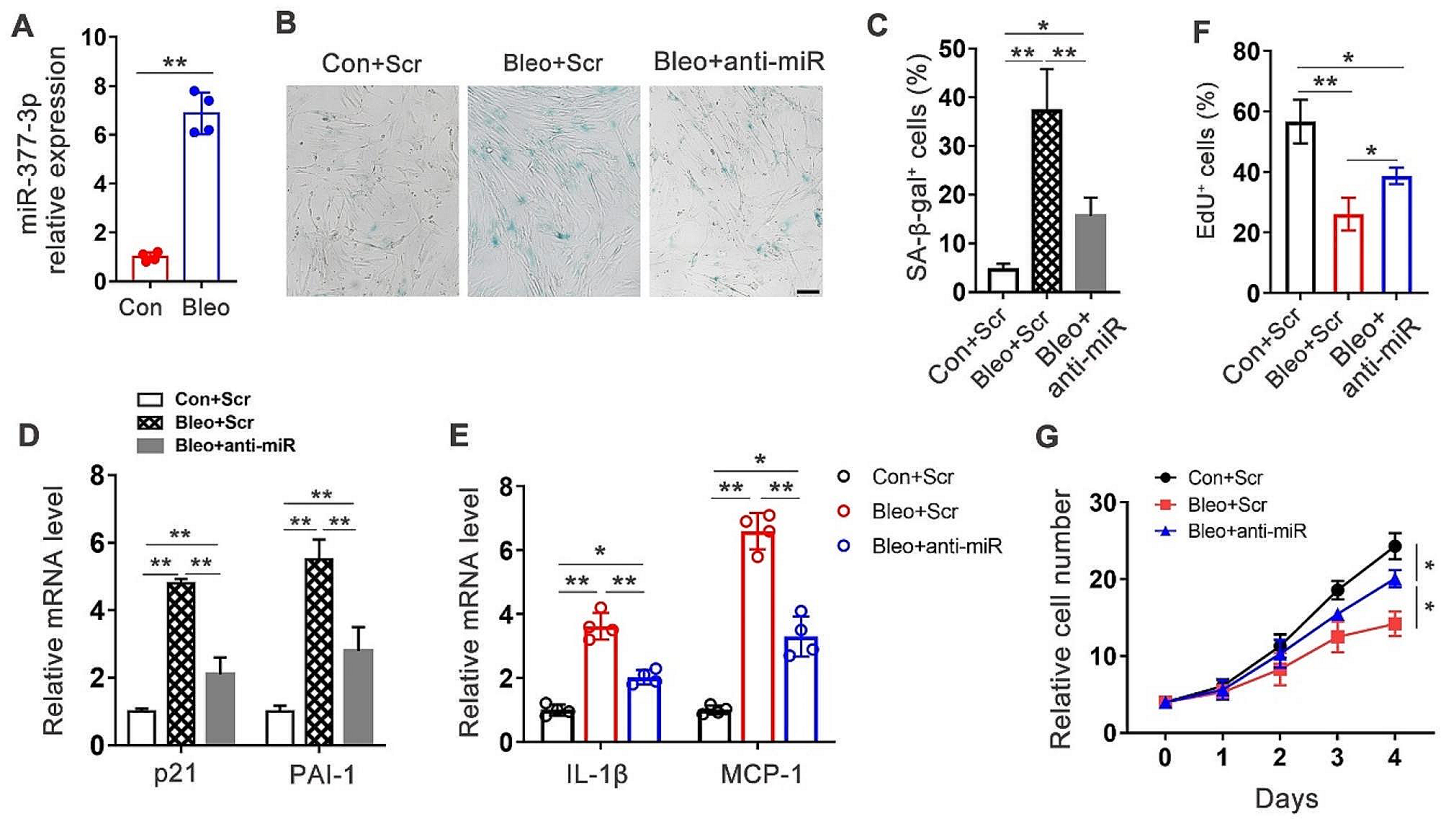
Knockdown of miR-377-3p attenuates bleomycin-induced senescence in lung fibroblasts. MRC-5 was treated by DMSO or bleomycin (0.01 U/mL). **(A)** The level of miR-377-3p was determined by real-time PCR (*n* = 4). (**B, C**) MRC-5 cells in 24-well plates were transfected with miR-377-3p specific antagomir (anti-miR) or scrambled (Scr) antagomir. SA-β-gal activity in the cells were determined by X-gal staining. Scale bar, 100 μm. **(D)** The mRNA levels of p21 and PAI-1 were determined by real-time PCR (*n* = 4). **(E)** Levels of IL-1β and MCP-1 were determined by real-time PCR (*n* = 4). **(F)** The cells were incubated with medium containing EdU for 6 h (*n* = 4). **(G)** Cells were trypsinized and counted at the indicated time points (*n* = 3)

Next, we investigated whether suppressing miR-377-3p may have the opposite impact on lung fibroblast senescence as overexpression. First, we induced MRC-5 cell senescence by bleomycin treatment, a wildly used cell model for senescence study [4]. The expression of miR-377-3p was dramatically elevated in bleomycin-induced senescent fibroblasts (Fig. 4A), implying a significant role of miR-377-3p in senescence regulation. More crucially, knockdown of miR-377-3p reduced the bleomycin-induced senescent phenotype, indicated by fewer SA-β-gal positive cells and lower expression of the senescence markers p21 and plasminogen activator inhibitor 1 (PAI-1) in miR-377-3p knockdown cells (Fig. 4B-D). The mRNA expression of SASP molecules, IL-1β and MCP-1, were also decreased upon miR-377-3p repression (Fig. 4E). In addition, we confirmed the anti-senescent effect of miR-377-3p inhibition by measurement of cell proliferation and growth. Both cell proliferation indicated by EdU incorporation and cell growth measured by cell counting based growth curve revealed that knockdown of miR-377-3p obviously interrupted bleomycin induced cell cycle arrest and promote cell proliferation (Fig. 4F and G).

### Mir-377-3p directly targets ZFP36L1 mRNA to induce senescent phenotype

**Fig. 5:**
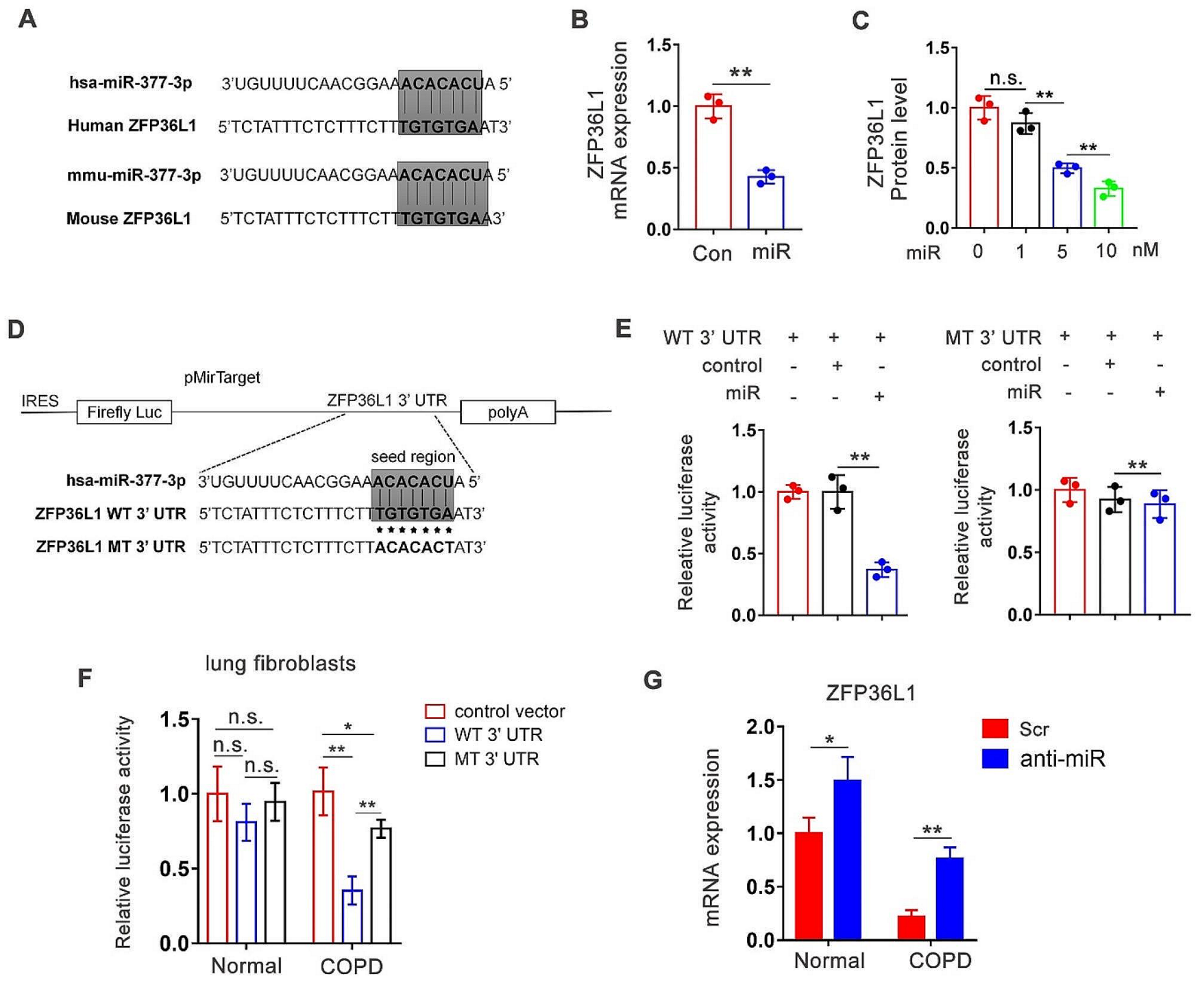
MiR-377-3p directly targets ZFP36L1 mRNA. **(A)** The seed region of miR-377-3p predicted to target the 3’ UTR of ZFP36L1 in human and mouse. **(B)** MRC-5 cells were transfected with control or miR-377-3p agomir (miR). The mRNA level of ZFP36L1 was measured by real-time PCR 24 h after transfection (*n* = 3). **(C)** Dose-dependent effect of miR-377-3p agomir on the abundance of ZFP36L1 protein. **(D)** Depiction of luciferase reporter construct for the WT 3’ UTR of the ZFP36L1 seed region and mutations. **(E)** 293T cells were transfected with 50 ng of either pMirTarget vector carrying WT or mutated 3’ UTR of ZFP36L1 with and without 50 nM miR-377-3p or control agomir. **(F)** The lung fibroblasts derived from normal or COPD lung tissues were transfected with luciferase reporter construct carrying either WT or MT 3’ UTR of ZFP36L1 (*n* = 3). **(G)** Primary lung fibroblasts were transfected with 50 nM miR-377-3p specific antagomir (anti-miR) or scrambled (Scr) antagomir. The level of RNA was measured 2 days after transfection (*n* = 3). Data are presented as means ± SD. n.s., not significant. *p < 0.05; **p < 0.01

MiRNAs generally bind to the 3’-UTR (untranslated region) of their target mRNAs and repress protein production by destabilizing the mRNA and/or translational silencing [5]. We performed in silico screen to find potential targets for miR-377-3p in the regulation of senescence in TargetScan [23] and miRDB database [24]. We selected the top-ranked candidate mRNAs and performed further screening based on the relevance to the cellular senescence process using information from existing research literature. We found that the seed region of both human and murine miR-377-3p matched 3’-UTR of ZFP36L1 mRNA very well (Fig. 5A). Overexpression of miR-377-3p agomir in MRC-5 suppressed ZFP36L1 mRNA expression obviously (Fig. 5B). The suppressive effect was dose dependent (Fig. 5C). To further characterize miR-377-3p’s direct regulation on ZFP36L1, we constructed luciferase expressing plasmids containing either wild-type (WT) or mutated (MT) 3’-UTR region of ZFP36L1 mRNA (Fig. 5D). The results showed that miR-377-3p agomir efficiently inhibited luciferase expression with WT 3’-UTR while having no impact with MT 3’-UTR, suggesting the regulation was depended on the sequence of seed region (Fig. 5E). We also introduced the luciferase plasmids into primary lung fibroblasts derived from normal subjects or COPD patients to test the influence of endogenous miR-377-3p on ZFP36L1 expression. As shown in Fig. 5F, luciferase expression with WT 3’-UTR was significantly repressed only in COPD fibroblasts, comparing to normal fibroblasts, indicating higher endogenous miR-377-3p under COPD condition. Furthermore, we suppressed miR-377-3p in primary lung fibroblasts and found that inhibition of miR-377-3p more effective in promoting the expression of ZFP36L1 in COPD fibroblasts (Fig. 5G).

### MiR-377-3p exacerbates fibroblast senescence through inhibition of ZFP36L1

**Fig. 6:**
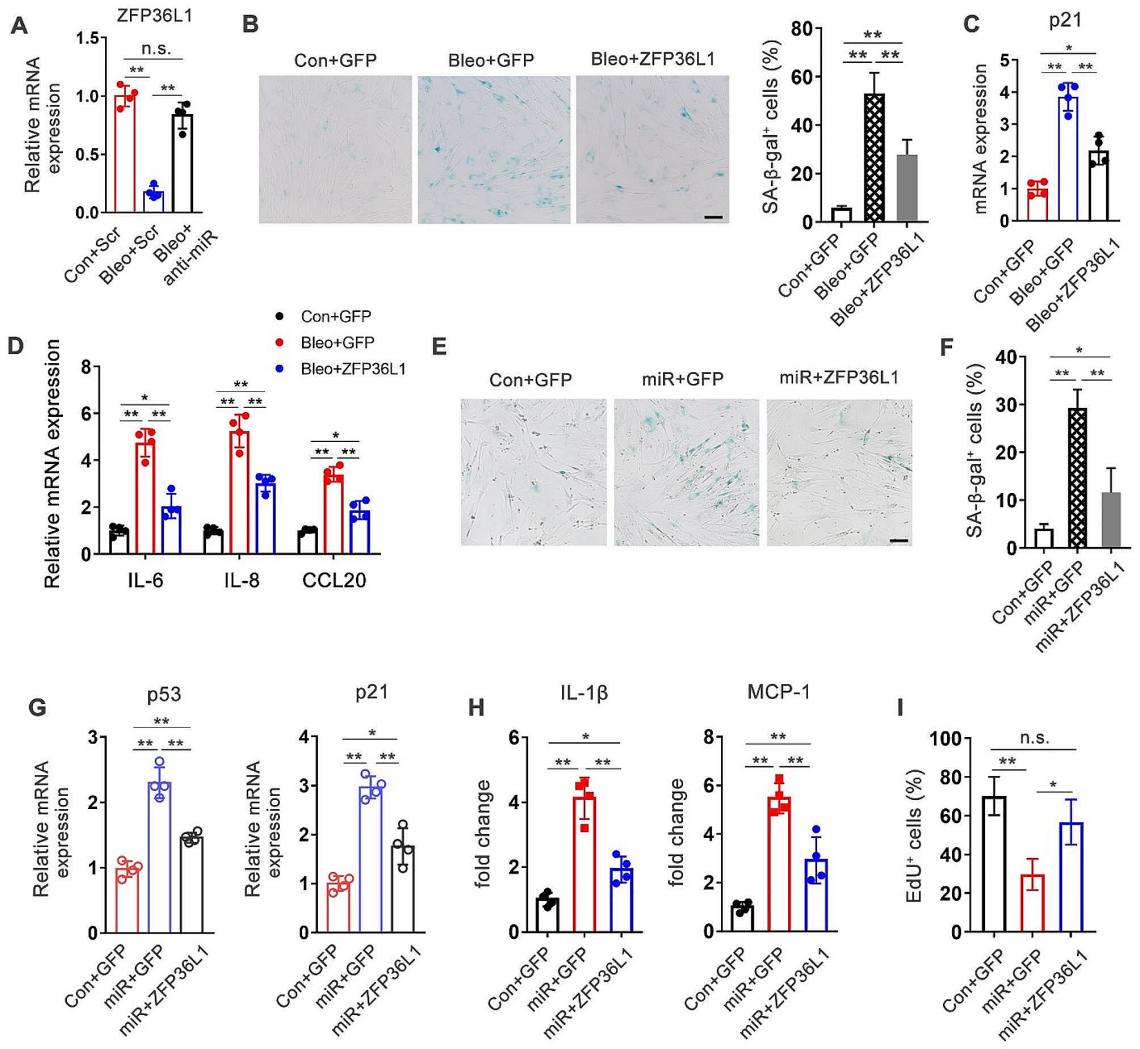
MiR-377-3p exacerbates lung fibroblast senescence through inhibition of ZFP36L1. **(A)** MRC-5 cells were transfected with 50 nM miR-377-3p specific antagomir (anti-miR) or scrambled (Scr) antagomir. After transfection, the cells were treated with DMSO or bleomycin. The mRNA level of ZFP36L1 was determined by real-time PCR. (B-D) MRC-5 cells were transduced with GFP (control) or ZFP36L1 expressing lentivirues. 48 h after transduction, the cells were treated with DMSO or bleomycin. SA-β-gal activity in the cells were determined by X-gal staining. **(B)** Left, representative images of SA-β-gal staining were shown. Scale bar, 100 μm. Right, SA-β-gal positive cells were counted from at least 10 field for each treatment. **(C)** The mRNA expression of p21 in MRC-5 cells were measured (*n* = 4). **(D)** The mRNA expression of SASP in MRC-5 cells were measured after bleomycin treatment. (E-I) MRC-5 cells were transduced with GFP (control) or ZFP36L1 expressing lentivirues. 1 day after transduction, the cells were transfected with 50 nM miR-377-3p or control agomir. The activity of SA-β-gal was measured by X-gal staining. **(E)** Representative images of SA-β-gal staining were shown. Scale bar, 100 μm. **(F)** SA-β-gal positive cells were counted from 10 fields for each treatment. **(G)** The mRNA expression of p53 and p21 in MRC-5 cells were measured by real-time PCR (*n* = 4). **(H)** The mRNA Levels of IL-1β and MCP-1 were determined by real-time PCR (*n* = 4). **(I)** The cells were incubated with medium containing EdU for 6 h (*n* = 4)

To further determine whether miR-377-3p regulates lung fibroblast senescence through inhibition of ZFP36L1, we suppressed miR-377-3p in bleomycin induced senescent fibroblasts. The results demonstrated that the inhibition of miR-377-3p largely restored the decrease of ZFP36L1 caused by bleomycin treatment (Fig. 6A). The introduction of ZFP36L1 in bleomycin-treated senescent fibroblasts showed a significant reduction in cellular senescence, as indicated by SA-β-gal staining and the expression of p21, p53 and SASP molecules (Fig. 6B-D, S3). Next, we expressed ZFP36L1 in miR-377-3p induced senescent cells. We found that both the proportion of SA-β-gal staining positive fibroblasts (Fig. 6E and F) and the expression of crucial regulators of senescence, p53 and p21 (Fig. 6G), were reduced after ZFP36L1 overexpression. The finding of diminished cellular senescence by ZFP36L1 expression also confirmed by measuring SASP expression (IL-1β and MCP-1) (Fig. 6H) and EdU incorporation (Fig. 6I). Taken together, these evidence suggested that the elevation of intracellular miR-377-3p triggered senescence by suppressing ZFP36L1 expression, at least in part.

## Discussion

In this study, we investigated the effects of miR-377-3p on the senescence of lung fibroblasts in vitro and COPD development in vivo, and tried to explore the underlying mechanism by which miR-377-3p modulates the pathology of COPD. Currently, most investigation on miR-377-3p have been carried out in tumor tissues or cells, and the conclusions are not consistent. The influence of miR-377-3p on the proliferation of tumour cells seems to rely on the context, displaying both oncogenic and tumour suppressive characteristics in different types of tumors [14–16]. These comparable findings indicate that the function of miR-377-3p may vary depending on the circumstances. In our preceding investigation, it was observed that miR-377-3p exhibited a noteworthy elevation in the bloodstream of individuals diagnosed with COPD. In this study, we primarily focused on the role of miR-377-3p in fibroblasts, given its concentrated expression in lung tissue and the significant contribution of fibroblasts to COPD. While the impact of senescent fibroblasts on idiopathic pulmonary fibrosis (IPF) has been extensively investigated, our understanding of the influence of senescent fibroblasts on COPD remains relatively limited. This study provides additional evidence regarding the involvement of senescent fibroblasts in the promotion of COPD. Notably, fibroblast senescence has the opposite impact on fibrotic illnesses; for example, senescence of hepatic stellate cells (fibroblasts in the liver) alleviates liver fibrosis [25, 26]. According to this viewpoint, the influence of fibroblast senescence on fibrotic illnesses is context-dependent. It is supposed that during the initial stages of fibrotic diseases, cellular senescence of fibroblasts may alleviate fibrosis deposition, whereas the accumulation of a substantial number of senescent fibroblasts may exacerbate fibrotic diseases. Further investigation of these matters is warranted in subsequent study.

ZFP36L1 is a Zn-finger protein that destabilizes mRNAs by binding to AU-rich elements (AREs) in their 3’ untranslated regions (3’-UTR). ZFP36L1 targets mRNAs of several cytokines for degradation [27]. Phosphorylation of ZFP36L1 by MAPKAPK2 inhibits its binding to mRNAs while stabilizing ZFP36L1 [28, 29]. Expression of the non-phosphorylatable mutant version of ZFP36L1 resistant to inactivation by MAPKAPK2, markedly reduced the levels of the SASP transcripts, rescued cell proliferation and inhibited cellular senescence [30]. In chronic myeloid leukemia (CML) cells, depletion of ZFP36L1 leads to decreased proliferation of CML cells upon treatment with tyrosine kinase inhibitor, while ZFP36L1 directly inhibits CDKN1A expression via post-transcriptional regulation [31].

The present investigation revealed that the upregulation of ZFP36L1 resulted in the inhibition of p21 expression, which is likely the underlying mechanism responsible for the attenuation of cellular senescence in lung fibroblasts by ZFP36L1. We suggests that the induction of senescence through miR-377-3p is attributed to its inhibitory effect on ZFP36L1, which subsequently triggers the expression of SASP component. The components of SASP exhibit a reinforcing impact on cells expressing miR-377-3p, as well as neighbouring cells, by means of autocrine and paracrine feedback mechanisms that induce senescence. Interestingly, the mTOR/MAPKAPK2/4EBP1/ZFP36L1 axis blunts both the pro-tumorigenic the tumour-suppressive effects of the SASP [30]. In contrast to cellular senescence induced by oncogene activation, our findings indicate a reduction in the expression of ZFP36L1 mRNA in cellular senescence induced by bleomycin. Comparable to the senescence induced by oncogene activation, ectopic expression of ZFP36L1 effectively reduced bleomycin-induced cellular senescence and SASP in lung fibroblasts. Further investigation is required to determine the existence of additional regulatory pathways for ZFP36L1 expression in senescent cells as well as the potential association between the activation state of mTOR and the expression of miR-377-3p.

Generally, our investigation enhances the comprehension of the involvement of miRNAs in the progression of COPD. On the basis of our previous study, we further explored the reasons for the increase of miR-377-3p in the blood of COPD patients and its regulatory role on the pathological progression of COPD. Of course, there are limitations in our study. We did not conduct an in-depth examination of the mechanism by which miR-377-3p was up-regulated in lung fibroblasts, which may pose some challenges to using miR-377-3p as an intervention target for COPD therapy. We will perform more in-depth research depending on the application needs in the follow-up study.

### Electronic supplementary material

Below is the link to the electronic supplementary material.

Supplementary Material 1

Supplementary Material 2

Supplementary Material 3

Supplementary Material 4

Supplementary Material 5

## Data Availability

The data that support the findings of this study are available from the corresponding author upon reasonable request.
