# Supplementary figures and images for: MicroRNA-377-3p exacerbates chronic obstructive pulmonary disease through suppressing ZFP36L1 expression and inducing lung fibroblast senescence
Source: Respir Res. 2024 Feb 5;25:67. doi: 10.1186/s12931-024-02696-3 (PMC10840170; doi:10.1186/s12931-024-02696-3)

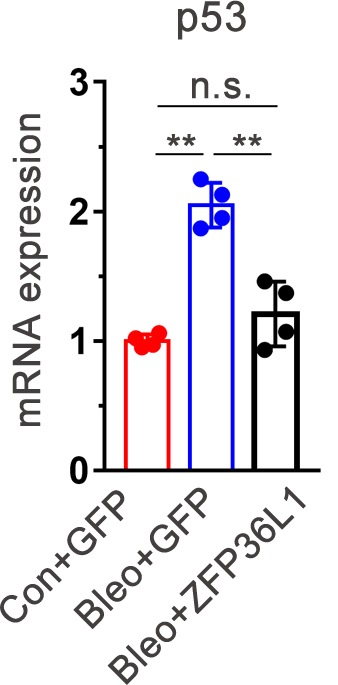

Supplement: Supplementary file 1 — Supplementary Material 1 [file 12931_2024_2696_MOESM1_ESM.jpg]

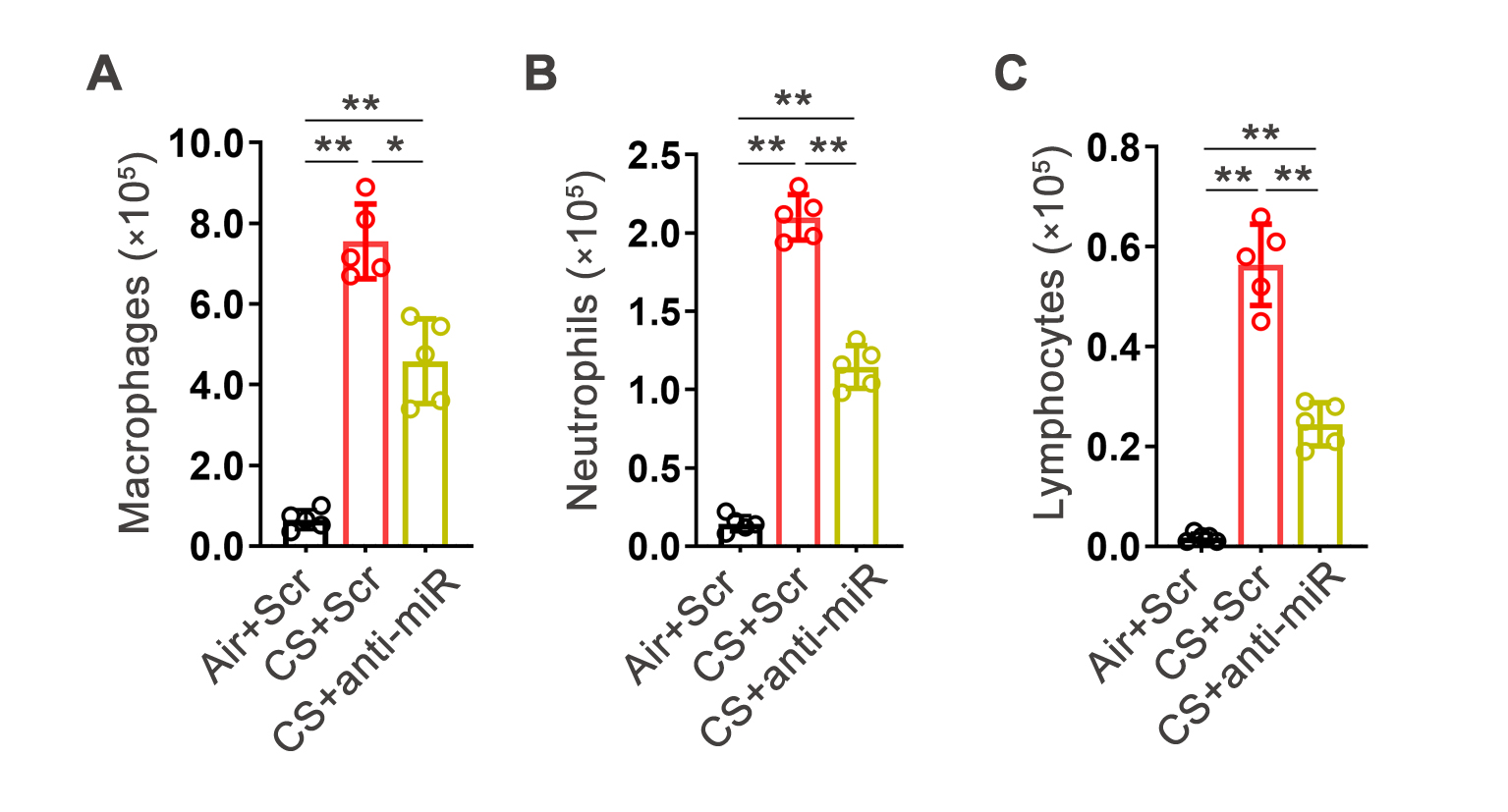

Supplement: Supplementary file 2 — Supplementary Material 2 [file 12931_2024_2696_MOESM2_ESM.jpg]

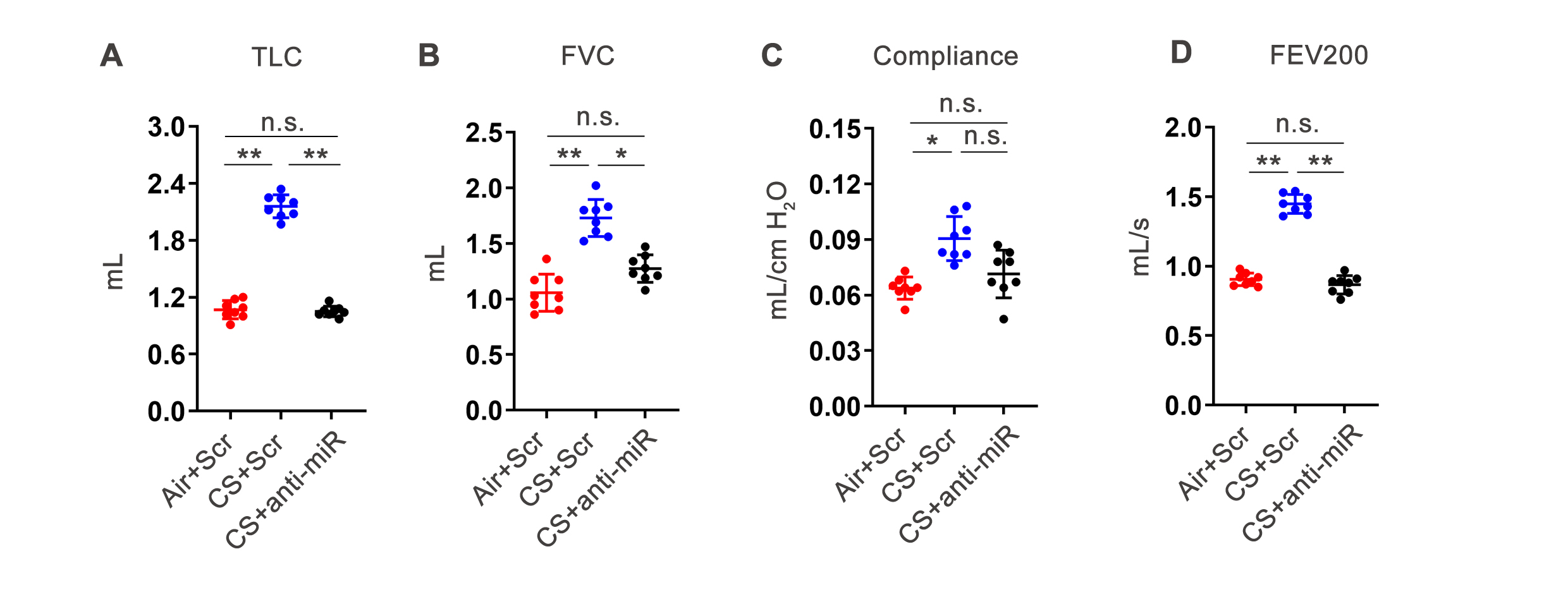

Supplement: Supplementary file 3 — Supplementary Material 3 [file 12931_2024_2696_MOESM3_ESM.jpg]

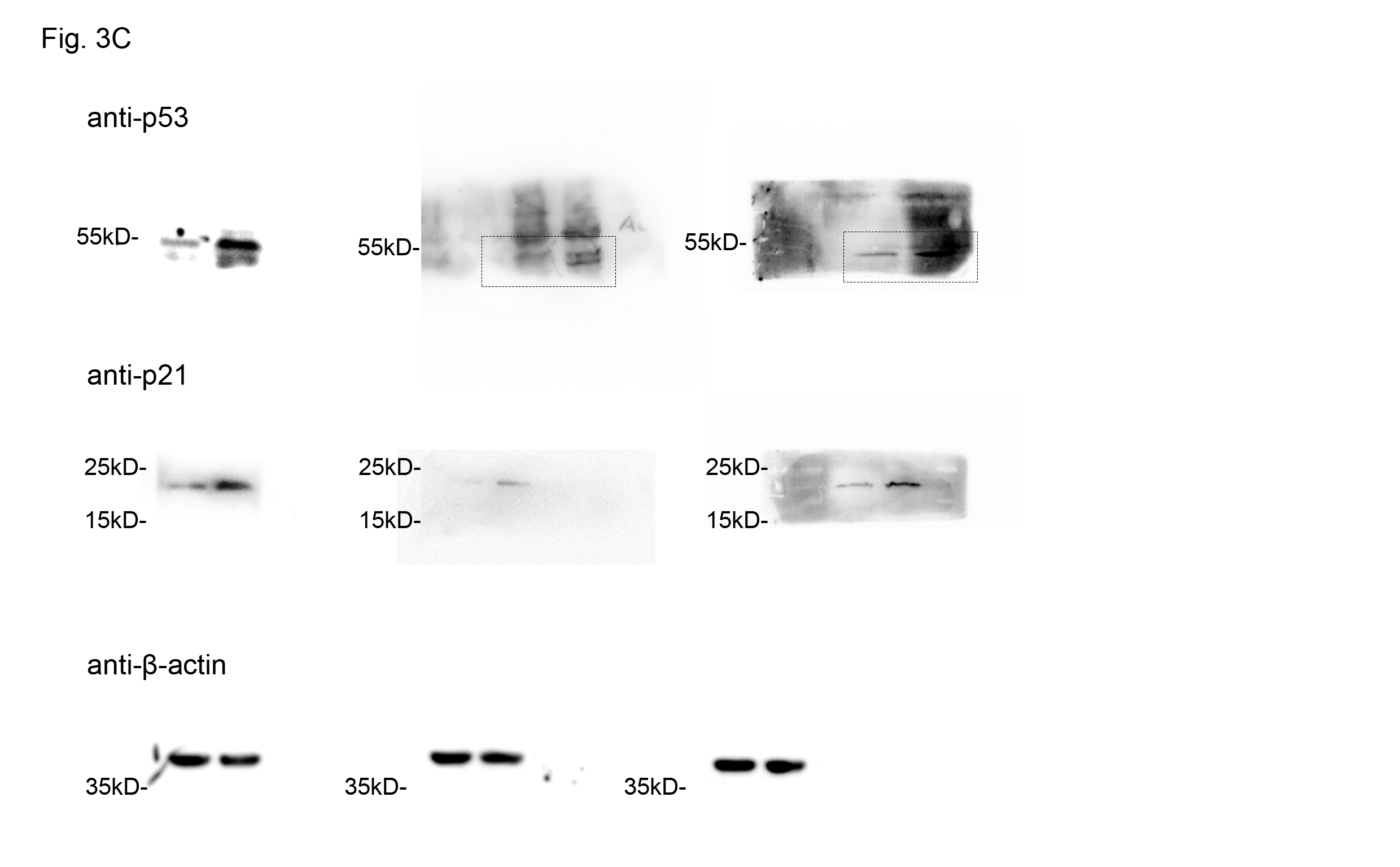

Supplement: Supplementary file 5 — Supplementary Material 5 [file 12931_2024_2696_MOESM5_ESM.png]
