# Supplementary material for: MicroRNA-377-3p exacerbates chronic obstructive pulmonary disease through suppressing ZFP36L1 expression and inducing lung fibroblast senescence
Source: Respir Res. 2024 Feb 5;25:67. doi: 10.1186/s12931-024-02696-3 (PMC10840170; doi:10.1186/s12931-024-02696-3)
Supplement: Supplementary file 4 — Supplementary Material 4 [file 12931_2024_2696_MOESM4_ESM.docx]

**Supplementary materials**

**Supplementary Figure S1. The change of BALF cellular composition after miR-377-3p antagomir intervention in CS induced experimental COPD mice.** The alveolar lavage fluid was collected and macrophage **(A)**, neutrophils **(B)**, and lymphocytes **(C)** were counted by a hemocytometer (n = 5). Values were expressed as mean ± SD. *p<0.05, **p<0.01.

**Supplementary Figure S2. Suppression of miR-377-3p improves pulmonary function in chronic smoking induced experimental COPD.** Pulmonary function, including total lung capacity (TLC), forced vital capacity (FVC), lung compliance (Compliance) and forced expiratory volume at 200 ms (FEV200) were measured (n = 8 mice per group). Values were expressed as mean ± SD. n.s., not significant; *p<0.05; **p<0.01.

**Supplementary Figure S3. Expression of ZFP36L1 reduces bleomycin induced p53 elevation in MRC-5 fibroblasts.** The mRNA expression of p53 in MRC-5 cells were measured by real-time PCR (n = 4). Values were expressed as mean ± SD. n.s., not significant; **p<0.01.

**Supplementary Figure S4** The full and non-adjusted images for cropped images of gels and blots.
